# Crystal structure of *trans*-di­aqua­(3,10-dimethyl-1,3,5,8,10,12-hexa­aza­cyclo­tetra­deca­ne)copper(II) pamoate

**DOI:** 10.1107/S2056989019003852

**Published:** 2019-04-02

**Authors:** Liudmyla V. Tsymbal, Irina L. Andriichuk, Vladimir B. Arion, Yaroslaw D. Lampeka

**Affiliations:** a L.V. Pisarzhevskii Institute of Physical Chemistry of the National Academy of Sciences of Ukraine, Prospekt Nauki 31, Kiev 03028, Ukraine; b Institute of Inorganic Chemistry of the University of Vienna, Wahringer Str. 42, 1090 Vienna, Austria

**Keywords:** crystal structure, aza­macrocyclic ligand, di­aza­cyclam, copper, pamoic acid, hydrogen bonds

## Abstract

The asymmetric unit of the title compound, *trans*-di­aqua­(3,10-dimethyl-1,3,5,8,10,12-hexa­aza­cyclo­tetra­decane-κ^4^ *N* ^1^,*N* ^5^,*N* ^8^,*N* ^12^)copper(II) 4,4′-methyl­ene­bis(3-hy­droxy­naphthalene-2-carboxyl­ate), [Cu(C_10_H_26_N_6_)(H_2_O)_2_](C_23_H_14_O_6_) {[Cu(*L*)(H_2_O)_2_](pam), where *L* = 3,10-dimethyl-1,3,5,8,10,12-hexa­aza­cyclo­tetra­decane and pam = dianion of pamoic acid} consists of two independent halves of the [Cu(*L*)(H_2_O)_2_]^2+^ cation and one di­carboxyl­ate anion. The Cu^II^ atoms, lying on inversion centres, are coordinated by the four secondary N atoms of the macrocyclic ligands and the mutually *trans* O atoms of the water mol­ecules in a tetra­gonally elongated octa­hedral geometry. The average equatorial Cu—N bond length is significantly shorter than the average axial Cu—O bond length [2.007 (10) and 2.486 (18) Å, respectively]. The macrocyclic ligand in the complex cations adopts the most energetically stable *trans*-III conformation. The complex cations and anions are connected *via* hydrogen-bonding inter­actions between the N—H groups of the macrocycles and the O—H groups of coordinated water mol­ecules as the proton donors and the O atoms of the carboxyl­ate as the proton acceptors into layers lying parallel to the (11) plane.

## Abstract 2

The complex cation of the title compound has a six-coordinate tetra­gonal–bipyramidal structure with four N atoms of the aza­macrocyclic ligand in the equatorial plane and two O atom of the water mol­ecules in the axial positions. In the crystal, the carb­oxy­lic groups of the non-coordinated dianion of pamoic acid accept N—H⋯O and O—H⋯O hydrogen bonds, forming sheets of ions lying parallel to the (1
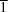
1) plane.

## Chemical context   

Coordination compounds of cyclam-like tetra­dentate aza­macrocyclic ligands (cyclam = 1,4,8,11-tetra­aza­cyclo­tetra­deca­ne) have attracted considerable attention because of their high thermodynamic stability, kinetic inertness, unusual redox properties and spectroscopic features (Melson, 1979 ▸; Yatsimirskii & Lampeka, 1985 ▸). Transition-metal complexes of this type of equatorial ligand possess two *trans* vacant sites in the axial positions and are suitable building blocks for the construction of metal–organic frameworks (MOFs) with potential applications in many areas including sorption, separation, gas storage, heterogeneous catalysis *etc* (Lampeka & Tsymbal, 2004 ▸; Suh & Moon, 2007 ▸; Suh *et al.*, 2012 ▸; Stackhouse & Ma, 2018 ▸; Lee & Moon, 2018 ▸). The Cu^II^ complexes of *N* ^3^,*N* ^10^-dialkyl-substituted di­aza­cyclam (di­aza­cyclam = 1,3,5,8,10,12-hexa­aza­cyclo­tetra­deca­ne), readily obtainable *via* template-directed Mannich condensation of bis­(ethyl­enedi­amine) complexes with formaldehyde and primary amines (Costisor & Linert, 2000 ▸), represent widespread systems in this kind of investigation.

Pamoic acid [4,4′-methyl­ene-bis­(3-hy­droxy­naphthalene-2-carb­oxy­lic acid), H_2_pam] is widely used as a counter-ion in pharmaceutical formulations (Du *et al.*, 2007 ▸ and references cited therein). This di­carb­oxy­lic acid is built from two naphthalene fragments, each bearing carb­oxy­lic and hydroxyl substituents and linked by a methyl­ene bridge. The combination of this potentially bridging ligand with a biometal complex (*e.g.* Cu^II^) could be a promising candidate for the construction of the Bio–MOFs attracting currently considerable attention (Cai *et al.*, 2019 ▸).
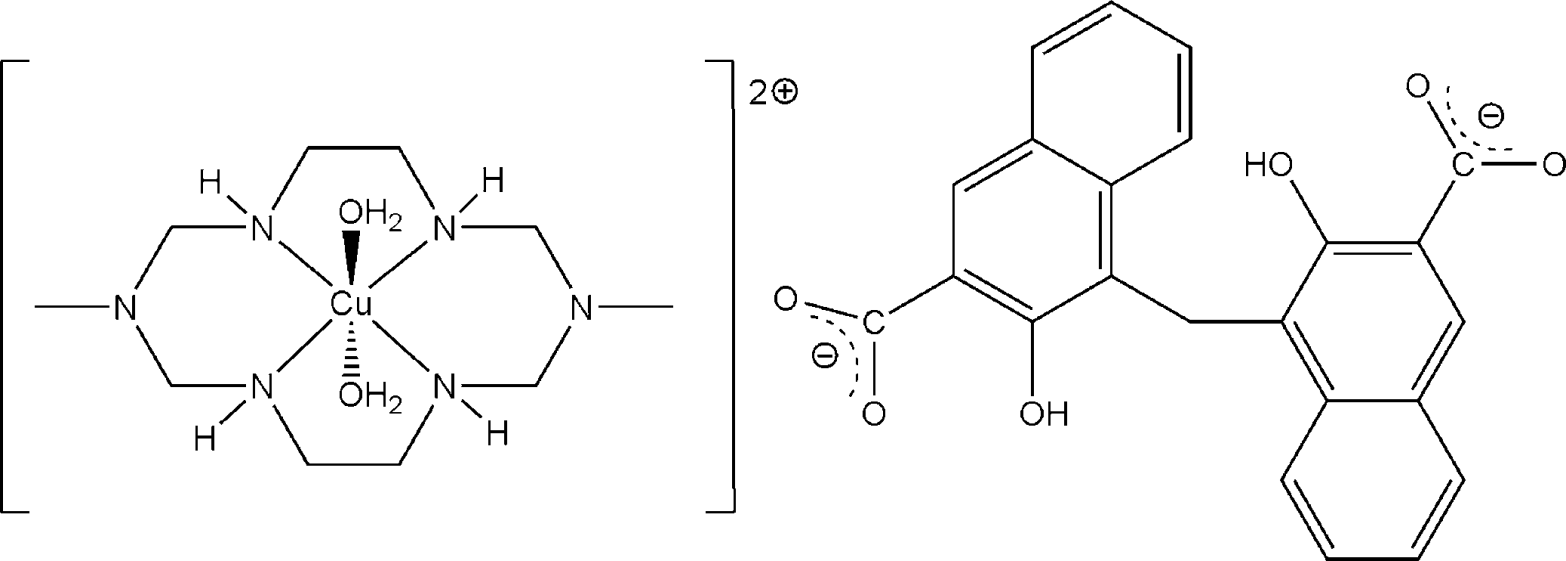


Here, we report the synthesis and the crystal structure of the title di­aqua–Cu^II^ complex with a di­aza­cyclam ligand and pamoate dianion, namely *trans*-di­aqua­(3,10-dimethyl-1,3,5,8,10,12-hexa­aza­cyclo­tetra­decane-κ^4^ *N^1^,N^5^,N^8^,N^12^*)copper(II) pamoate, [Cu*L*(H_2_O)_2_](pam), (I).

## Structural commentary   

**Figure 1:**
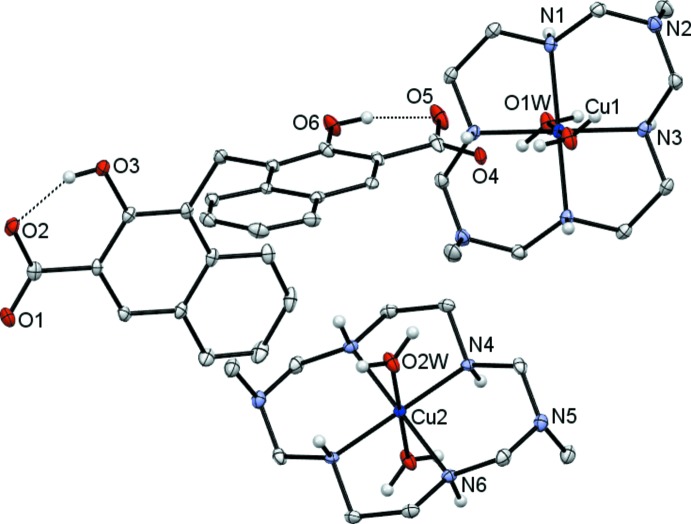
View of the mol­ecular structure of (I), showing the partial atom-labelling scheme, with thermal displacement ellipsoids drawn at the 30% probability level. H atoms at carbon atoms have been omitted for clarity. Intra-anion hydrogen-bonding inter­actions are shown as dashed lines.

The title compound (I) contains two crystallographically independent centrosymmetric complex cations. Each Cu^II^ ion lies on an inversion centre and is coordinated in the equatorial plane by four secondary amine N atoms of the aza­macrocyclic ligand in a square-planar fashion, and by two O atoms from the water mol­ecules in the axial positions, resulting in a tetra­gonally distorted octa­hedral geometry (Table 1 ▸, Fig. 1 ▸).

The CuN_4_ fragments in (I) are strictly planar; at the same time they display some rhombic distortion. In particular, the Cu1—N3 and Cu2—N4 distances [av. 2.000 (1) Å] are shorter than those for Cu1—N1 and Cu2—N6 bonds [av. 2.014 (3) Å]. The axial bonds Cu—O*W* [av. 2.486 (17) Å] are longer than the equatorial bonds, which can be attributed to a large Jahn–Teller distortion. The coordinated macrocyclic ligand in both cations adopts the most energetically favourable *trans*-III (*R*,*R*,*S*,*S*) conformation (Bosnich *et al.*, 1965 ▸) with the five- and six-membered chelate rings in *gauche* and *chair* conformations, respectively. The bite angles in the five- and six-membered chelate rings equal 86.53 (8) and 93.47 (8)°, respectively. The methyl substituents at the distal nitro­gen atoms in the six-membered chelate rings are axially oriented. Therewith, the C—N—C angles at non-coordinated nitro­gen atoms (*ca* 115°) are larger than the canonical value for an *sp^3^*-hybridized nitro­gen atom (109°), thus indicating their partial *sp^2^* character.

The V-shaped pamoate dianion is fully deprotonated to counterbalance the charge of the complex unit and possesses a twisted conformation with the joint angle between the naphthalene rings being 115.6 (2)° and the angle between the mean planes of naphthalene fragments being 88.6 (2)°. The carb­oxy­lic groups adopt a transoid configuration to minimize unfavorable steric hindrance (Du *et al.*, 2007 ▸). The C—O bond lengths in each carb­oxy­lic group are somewhat different [1.248 (3) *versus* 1.271 (3) and 1.245 (3) *versus* 1.279 (4) Å for the O1—C11—O2 and O4—C22—O5 fragments, respectively], thus indicating their incomplete delocalization. As expected, each hy­droxy­lic group exhibits a strong intra-anion O—H⋯O bond with the adjacent carboxyl oxygen (*D*⋯*A* distances *ca* 2.5 Å; Table 2 ▸).

## Supra­molecular features   

**Figure 2:**
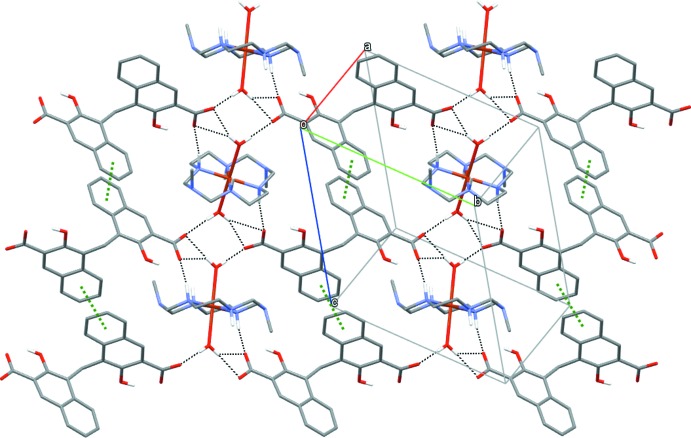
Sheets of complex mol­ecules parallel to the (1
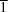
1) plane. Supra­molecular inter­actions are shown as dashed lines (black for hydrogen bonding and green for π–π inter­actions). H atoms at carbon atoms and intra-anion hydrogen bonds are not shown.

Each carboxyl­ate group of the pamoate anion acts as a proton acceptor by the formation of N—H⋯O hydrogen bonds with adjacent secondary amine groups of the aza­macrocyclic ligand and bifurcated O*W*—H⋯(O,O) hydrogen bonds with a coordinated water mol­ecule of the same cation (Fig. 2 ▸ and Table 2 ▸). Additionally, the benzene fragments of the naphthalene rings are involved in two kinds of inter­molecular π–π inter­actions [inter­planar separation of 3.470 and 3.717 Å; centroid-to-centroid distances of 3.8996 (15) and 4.2107 (15) Å, respectively] (Fig. 2 ▸). These supra­molecular inter­actions (Steed & Atwood, 2009 ▸) generate sheets of inter­acting ions parallel to (1
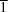
1), and additional N1—H1⋯O3 contacts and C—H⋯O inter­actions link these sheets into a three-dimensional network.

## Database survey   

A search of the Cambridge Structural Database (CSD, version 5.39, last update August 2018; Groom *et al.*, 2016 ▸) indicated that 65 Cu^II^ complexes of *N* ^3^,*N* ^10^-disubstituted di­aza­cyclams with various alkyl pendant groups have been reported and the majority of them were investigated as building blocks for supra­molecular chemistry. Among them, eight hits deal with a di­aqua aza­macrocyclic Cu^II^ cation. Surprisingly, only one structure with the dimethyl-substituted macrocycle *L* has been reported, *i.e.* [Cu(*L*)](ClO_4_)_2_ (LAWXIR; Zhang *et al.*, 2005 ▸) and the title compound (I) is the first example of a [Cu(*L*)(H_2_O)_2_]^2+^ cation described so far.

A search for pamoic acid gave 97 hits, only four of which concern compounds consisting of uncoordinated pamoate dianion and metal complex cations, *i.e.*, [*M*(H_2_O)_2_(phen)_2_](pam)·H_2_O [*M* = Zn^II^ (MEBGOQ), Mn^II^ (SIQDOM), Cd^II^ (YOLDEJ), phen = phenanthroline] and [Mn(H_2_O)_4_(DMF)_2_](pam) (SIQCOL) (Ma *et al.*, 2006 ▸; Du *et al.*, 2007 ▸; Shi *et al.*, 2008 ▸). Except for nine hits concerning the non-deprotonated pamoic acid, all other 84 structures are coordination polymers, thus demonstrating the availability of the pamoic acid anion for the design of MOFs.

## Synthesis and crystallization   

All chemicals and solvents used in this work were purchased from Sigma–Aldrich and used without further purification. The starting complex, [Cu(*L*)](ClO_4_)_2_, was prepared by a method reported in the literature (Suh & Kang, 1988 ▸). The title compound (I) was prepared as follows. To a water/DMF solution (1/3 by volume, 5 ml) of [Cu(*L*)](ClO_4_)_2_ (123 mg, 0.25 mmol) was added a DMF solution (10 ml) containing pamoic acid (97 mg, 0.25 mmol) and 0.2 ml of tri­ethyl­amine. A pink precipitate was formed in three days. This was filtered off, washed with a small amount of DMF and diethyl ether, and dried in air. Yield: 82 mg (46%). Analysis calculated for C_33_H_44_N_6_CuO_8_: C 55.33, H 6.19, N 11.73%. Found: C 55.42, H 6.24, N 11.62%. Single crystals suitable for X-ray diffraction analysis were selected from the sample resulting from the synthesis.

**Safety note**: Perchlorate salts of metal complexes are potentially explosive and should be handled with care.

## Refinement   

Crystal data, data collection and structure refinement details are summarized in Table 3 ▸. All H atoms were placed in geometrically idealized positions and constrained to ride on their parent atoms, with C—H distances of 0.95 (ring H atoms) or 0.98–0.99 Å (open-chain H atoms), N—H distance of 1.0 Å, hydroxyl O—H distance of 0.84 Å and aqua O—H distance of 0.86 Å with *U*_iso_(H) values of 1.2 or 1.5*U*_eq_ times that of the parent atoms.

## Supplementary Material

Crystal structure: contains datablock(s) I. DOI: 10.1107/S2056989019003852/hb7810sup1.cif

Structure factors: contains datablock(s) I. DOI: 10.1107/S2056989019003852/hb7810Isup2.hkl

CCDC reference: 1904400

Additional supporting information:  crystallographic information; 3D view; checkCIF report

## Figures and Tables

**Table 1:** Selected bond lengths (Å)

Cu1—N3	2.000 (2)	Cu2—N4	1.9987 (19)
Cu1—N1	2.017 (2)	Cu2—N6	2.0113 (19)
Cu1—O1w	2.5033 (19)	Cu2—O2w	2.4681 (18)

**Table 2:** Hydrogen-bond geometry (Å, °)

*D*—H⋯*A*	*D*—H	H⋯*A*	*D*⋯*A*	*D*—H⋯*A*
N1—H1⋯O3^i^	1.00	2.50	3.272 (3)	134
N3—H3⋯O5	1.00	1.89	2.836 (3)	156
N4—H4⋯O2^i^	1.00	1.90	2.822 (3)	152
O3—H3*C*⋯O2	0.84	1.75	2.502 (2)	148
O6—H6*C*⋯O5	0.84	1.75	2.514 (3)	150
O1*W*—H1*WA*⋯O1^ii^	0.86	1.88	2.746 (2)	178
O1*W*—H1*WB*⋯O4	0.86	2.31	3.136 (3)	162
O1*W*—H1*WB*⋯O5	0.86	2.41	3.087 (3)	136
O2*W*—H2*WA*⋯O1^i^	0.86	2.05	2.901 (2)	169
O2*W*—H2*WA*⋯O2^i^	0.86	2.61	3.280 (2)	136
O2*W*—H2*WB*⋯O4^iii^	0.86	1.88	2.743 (3)	176
C2—H2*B*⋯O1^iv^	0.99	2.48	3.435 (3)	162
C5—H5*B*⋯O2^i^	0.98	2.45	3.316 (3)	147

Symmetry codes: (i) 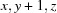; (ii) 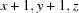; (iii) 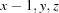; (iv) 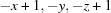.

**Table 3:** Experimental details

Crystal data	
Chemical formula	[Cu(C_10_H_26_N_6_)(H_2_O)_2_]C_23_H_14_O_6_
*M*_r_	716.28
Crystal system, space group	Triclinic, *P* 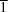
Temperature (K)	100
*a*, *b*, *c* (Å)	9.8877 (6), 12.1406 (7), 14.5760 (9)
α, β, γ (°)	71.594 (3), 81.128 (3), 88.249 (3)
*V* (Å^3^)	1640.06 (17)
*Z*	2
Radiation type	Mo *K*α
μ (mm^−1^)	0.73
Crystal size (mm)	0.20 × 0.18 × 0.12

Data collection	
Diffractometer	Bruker APEXII CCD
Absorption correction	Multi-scan (*SADABS*; Bruker, 2007 ▸)
*T*_min_, *T*_max_	0.868, 0.918
No. of measured, independent and observed [*I* > 2σ(*I*)] reflections	52866, 6401, 4540
*R*_int_	0.080
(sin θ/λ)_max_ (Å^−1^)	0.617

Refinement	
*R*[*F* ^2^ > 2σ(*F* ^2^)], *wR*(*F* ^2^), *S*	0.040, 0.090, 1.03
No. of reflections	6401
No. of parameters	438
No. of restraints	6
H-atom treatment	H-atom parameters constrained
Δρ_max_, Δρ_min_ (e Å^−3^)	0.34, −0.39

Computer programs: *APEX2* and *SAINT* (Bruker, 2007 ▸), *SHELXS2014* (Sheldrick, 2015*a* ▸), *SHELXL2014* (Sheldrick, 2015*b* ▸), *Mercury* (Macrae *et al.*, 2008 ▸) and *publCIF* (Westrip, 2010 ▸).

## supplementary crystallographic information

### Crystal data

**Table:**

[Cu(C_10_H_26_N_6_)(H_2_O)_2_]C_23_H_14_O_6_	*Z* = 2
*M**_r_* = 716.28	*F*(000) = 754
Triclinic, *P*1	*D*_x_ = 1.450 Mg m^−^^3^
*a* = 9.8877 (6) Å	Mo *K*α radiation, λ = 0.71073 Å
*b* = 12.1406 (7) Å	Cell parameters from 8230 reflections
*c* = 14.5760 (9) Å	θ = 2.4–25.6°
α = 71.594 (3)°	µ = 0.73 mm^−^^1^
β = 81.128 (3)°	*T* = 100 K
γ = 88.249 (3)°	Block, pink
*V* = 1640.06 (17) Å^3^	0.20 × 0.18 × 0.12 mm

### Data collection

**Table:**

Bruker APEXII CCD diffractometer	4540 reflections with *I* > 2σ(*I*)
Radiation source: fine-focus sealed tube	*R*_int_ = 0.080
φ and ω scans	θ_max_ = 26.0°, θ_min_ = 1.9°
Absorption correction: multi-scan (SADABS; Bruker, 2007)	*h* = −12→12
*T*_min_ = 0.868, *T*_max_ = 0.918	*k* = −14→14
52866 measured reflections	*l* = −17→17
6401 independent reflections	

### Refinement

**Table:**

Refinement on *F*^2^	6 restraints
Least-squares matrix: full	Hydrogen site location: mixed
*R*[*F*^2^ > 2σ(*F*^2^)] = 0.040	H-atom parameters constrained
*wR*(*F*^2^) = 0.090	*w* = 1/[σ^2^(*F*_o_^2^) + (0.0378*P*)^2^ + 0.6293*P*] where *P* = (*F*_o_^2^ + 2*F*_c_^2^)/3
*S* = 1.03	(Δ/σ)_max_ < 0.001
6401 reflections	Δρ_max_ = 0.34 e Å^−^^3^
438 parameters	Δρ_min_ = −0.39 e Å^−^^3^

### Special details

**Table:**

Geometry. All esds (except the esd in the dihedral angle between two l.s. planes) are estimated using the full covariance matrix. The cell esds are taken into account individually in the estimation of esds in distances, angles and torsion angles; correlations between esds in cell parameters are only used when they are defined by crystal symmetry. An approximate (isotropic) treatment of cell esds is used for estimating esds involving l.s. planes.

### Fractional atomic coordinates and isotropic or equivalent isotropic displacement parameters (Å^2^)

**Table:**

	*x*	*y*	*z*	*U*_iso_*/*U*_eq_	
Cu1	1.0000	0.5000	0.5000	0.01499 (12)	
Cu2	0.5000	0.5000	0.0000	0.01582 (12)	
N1	0.8280 (2)	0.58002 (17)	0.45737 (15)	0.0199 (5)	
H1	0.8412	0.6050	0.3843	0.024*	
N2	0.7064 (2)	0.40151 (18)	0.47031 (15)	0.0239 (5)	
N3	0.9443 (2)	0.34915 (17)	0.48721 (14)	0.0194 (5)	
H3	0.9644	0.3562	0.4162	0.023*	
C4	0.8207 (3)	0.6863 (2)	0.48671 (19)	0.0223 (6)	
H4A	0.7880	0.6669	0.5578	0.027*	
H4B	0.7566	0.7417	0.4510	0.027*	
C1	0.6994 (3)	0.5083 (2)	0.49364 (19)	0.0239 (6)	
H1A	0.6785	0.4901	0.5656	0.029*	
H1B	0.6232	0.5545	0.4650	0.029*	
C2	0.7977 (3)	0.3164 (2)	0.52012 (19)	0.0235 (6)	
H2A	0.7810	0.2416	0.5096	0.028*	
H2B	0.7761	0.3046	0.5912	0.028*	
C3	1.0361 (3)	0.2604 (2)	0.53813 (19)	0.0224 (6)	
H3A	1.0350	0.1910	0.5164	0.027*	
H3B	1.0057	0.2365	0.6097	0.027*	
C5	0.7162 (3)	0.4154 (2)	0.3666 (2)	0.0332 (7)	
H5A	0.7205	0.3388	0.3573	0.050*	
H5B	0.6356	0.4560	0.3418	0.050*	
H5C	0.7991	0.4607	0.3309	0.050*	
N6	0.5743 (2)	0.35155 (17)	0.08194 (14)	0.0190 (5)	
H6	0.6453	0.3239	0.0379	0.023*	
N4	0.6263 (2)	0.58227 (17)	0.05243 (14)	0.0169 (5)	
H4	0.5701	0.6038	0.1072	0.020*	
N5	0.5995 (2)	0.77617 (18)	−0.06320 (15)	0.0234 (5)	
C7	0.4716 (3)	0.2553 (2)	0.13005 (19)	0.0244 (6)	
H7A	0.5187	0.1862	0.1671	0.029*	
H7B	0.4038	0.2788	0.1776	0.029*	
C8	0.6454 (3)	0.3839 (2)	0.15230 (18)	0.0229 (6)	
H8A	0.7082	0.3217	0.1807	0.028*	
H8B	0.5780	0.3952	0.2061	0.028*	
C9	0.7253 (3)	0.4959 (2)	0.09671 (19)	0.0222 (6)	
H9A	0.7709	0.5232	0.1418	0.027*	
H9B	0.7963	0.4836	0.0452	0.027*	
C6	0.6926 (3)	0.6918 (2)	−0.01746 (19)	0.0223 (6)	
H6A	0.7471	0.7270	0.0181	0.027*	
H6B	0.7568	0.6723	−0.0690	0.027*	
C10	0.5140 (3)	0.8320 (2)	−0.0004 (2)	0.0290 (7)	
H10A	0.4544	0.8878	−0.0391	0.043*	
H10B	0.4577	0.7730	0.0525	0.043*	
H10C	0.5724	0.8727	0.0276	0.043*	
C11	0.4511 (3)	−0.3050 (2)	0.24922 (17)	0.0210 (6)	
C12	0.4904 (3)	−0.1995 (2)	0.27246 (17)	0.0169 (6)	
C13	0.6302 (3)	−0.1758 (2)	0.27572 (17)	0.0188 (6)	
C14	0.6685 (3)	−0.0783 (2)	0.29585 (17)	0.0181 (6)	
C15	0.5639 (3)	−0.0083 (2)	0.32585 (17)	0.0191 (6)	
C16	0.5905 (3)	0.0822 (2)	0.36370 (18)	0.0250 (6)	
H16	0.6820	0.0984	0.3687	0.030*	
C17	0.4868 (3)	0.1461 (2)	0.3929 (2)	0.0327 (7)	
H17	0.5068	0.2043	0.4199	0.039*	
C18	0.3508 (3)	0.1271 (2)	0.3835 (2)	0.0346 (7)	
H18	0.2799	0.1739	0.4022	0.041*	
C19	0.3211 (3)	0.0418 (2)	0.34780 (19)	0.0275 (7)	
H19	0.2292	0.0299	0.3409	0.033*	
C20	0.4247 (3)	−0.0295 (2)	0.32067 (17)	0.0200 (6)	
C21	0.3934 (3)	−0.1235 (2)	0.29063 (17)	0.0187 (6)	
H21	0.3018	−0.1346	0.2827	0.022*	
C22	1.0315 (3)	0.3478 (3)	0.2154 (2)	0.0297 (7)	
C23	0.9735 (2)	0.2584 (2)	0.17946 (19)	0.0199 (6)	
C24	0.9275 (3)	0.1467 (2)	0.24487 (18)	0.0216 (6)	
C25	0.8696 (2)	0.0657 (2)	0.21366 (17)	0.0178 (6)	
C26	0.8648 (2)	0.0906 (2)	0.11176 (18)	0.0168 (5)	
C27	0.8225 (2)	0.0078 (2)	0.07126 (18)	0.0203 (6)	
H27	0.7934	−0.0675	0.1132	0.024*	
C28	0.8227 (3)	0.0346 (2)	−0.02714 (19)	0.0250 (6)	
H28	0.7950	−0.0227	−0.0526	0.030*	
C29	0.8634 (3)	0.1459 (2)	−0.09125 (19)	0.0285 (7)	
H29	0.8607	0.1640	−0.1593	0.034*	
C30	0.9069 (3)	0.2277 (2)	−0.05578 (19)	0.0253 (6)	
H30	0.9349	0.3025	−0.0995	0.030*	
C31	0.9107 (2)	0.2024 (2)	0.04560 (18)	0.0189 (6)	
C32	0.9621 (2)	0.2839 (2)	0.08278 (19)	0.0203 (6)	
H32	0.9897	0.3589	0.0393	0.024*	
C33	0.8189 (3)	−0.0505 (2)	0.28762 (18)	0.0208 (6)	
H33A	0.8729	−0.1128	0.2698	0.025*	
H33B	0.8376	−0.0519	0.3528	0.025*	
O1	0.32781 (19)	−0.32344 (15)	0.24857 (13)	0.0262 (4)	
O2	0.54697 (19)	−0.37048 (15)	0.23045 (13)	0.0253 (4)	
O3	0.72922 (18)	−0.25007 (14)	0.25674 (13)	0.0242 (4)	
H3C	0.6944	−0.3007	0.2389	0.036*	
O4	1.06005 (19)	0.44690 (16)	0.15717 (16)	0.0377 (5)	
O5	1.0477 (2)	0.31618 (19)	0.30534 (15)	0.0416 (6)	
O6	0.9387 (2)	0.11959 (17)	0.34181 (13)	0.0319 (5)	
H6C	0.9751	0.1758	0.3514	0.048*	
O1W	1.07942 (19)	0.56232 (16)	0.31930 (13)	0.0303 (5)	
H1WA	1.1574	0.5988	0.2985	0.045*	
H1WB	1.0603	0.5205	0.2849	0.045*	
O2W	0.32694 (18)	0.51823 (16)	0.13556 (13)	0.0289 (5)	
H2WA	0.3381	0.5611	0.1708	0.043*	
H2WB	0.2426	0.4986	0.1394	0.043*	

### Atomic displacement parameters (Å^2^)

**Table:**

	*U*^11^	*U*^22^	*U*^33^	*U*^12^	*U*^13^	*U*^23^
Cu1	0.0177 (2)	0.0117 (2)	0.0152 (2)	−0.00018 (18)	−0.00184 (18)	−0.00397 (17)
Cu2	0.0159 (2)	0.0148 (2)	0.0163 (2)	−0.00108 (18)	−0.00325 (18)	−0.00389 (18)
N1	0.0235 (12)	0.0179 (11)	0.0181 (11)	−0.0009 (9)	−0.0016 (9)	−0.0062 (9)
N2	0.0277 (13)	0.0216 (12)	0.0224 (12)	−0.0033 (10)	−0.0082 (10)	−0.0044 (10)
N3	0.0248 (12)	0.0166 (11)	0.0157 (10)	0.0005 (9)	−0.0033 (9)	−0.0036 (9)
C4	0.0272 (15)	0.0188 (14)	0.0218 (14)	0.0048 (11)	−0.0041 (12)	−0.0075 (11)
C1	0.0198 (15)	0.0239 (15)	0.0252 (14)	0.0004 (11)	−0.0018 (11)	−0.0043 (12)
C2	0.0266 (16)	0.0189 (14)	0.0235 (14)	−0.0052 (12)	−0.0056 (12)	−0.0030 (12)
C3	0.0297 (16)	0.0142 (13)	0.0221 (14)	0.0029 (11)	−0.0045 (12)	−0.0040 (11)
C5	0.0401 (18)	0.0313 (16)	0.0332 (16)	−0.0010 (14)	−0.0185 (14)	−0.0109 (13)
N6	0.0192 (12)	0.0179 (11)	0.0183 (11)	0.0017 (9)	−0.0026 (9)	−0.0038 (9)
N4	0.0126 (11)	0.0189 (11)	0.0190 (11)	0.0020 (9)	−0.0014 (9)	−0.0065 (9)
N5	0.0256 (13)	0.0190 (12)	0.0239 (12)	−0.0029 (10)	0.0006 (10)	−0.0061 (10)
C7	0.0265 (16)	0.0194 (14)	0.0215 (14)	−0.0014 (12)	0.0010 (12)	−0.0003 (11)
C8	0.0223 (15)	0.0265 (15)	0.0206 (13)	0.0054 (12)	−0.0069 (11)	−0.0068 (12)
C9	0.0177 (14)	0.0282 (15)	0.0240 (14)	0.0044 (12)	−0.0077 (11)	−0.0111 (12)
C6	0.0169 (15)	0.0235 (15)	0.0260 (14)	−0.0055 (12)	0.0035 (12)	−0.0100 (12)
C10	0.0319 (17)	0.0210 (15)	0.0339 (16)	0.0007 (12)	−0.0008 (13)	−0.0106 (13)
C11	0.0306 (17)	0.0190 (14)	0.0108 (12)	−0.0038 (13)	−0.0002 (11)	−0.0021 (11)
C12	0.0226 (15)	0.0136 (13)	0.0131 (12)	−0.0037 (11)	0.0000 (10)	−0.0030 (10)
C13	0.0231 (15)	0.0154 (13)	0.0154 (13)	0.0013 (11)	0.0009 (11)	−0.0035 (11)
C14	0.0234 (15)	0.0148 (13)	0.0126 (12)	−0.0040 (11)	−0.0018 (11)	0.0004 (10)
C15	0.0287 (16)	0.0134 (13)	0.0112 (12)	−0.0029 (11)	0.0016 (11)	−0.0002 (10)
C16	0.0308 (16)	0.0223 (14)	0.0195 (14)	−0.0076 (12)	0.0022 (12)	−0.0050 (12)
C17	0.050 (2)	0.0204 (15)	0.0275 (15)	−0.0080 (14)	0.0092 (14)	−0.0135 (13)
C18	0.0360 (19)	0.0261 (16)	0.0377 (17)	0.0003 (14)	0.0134 (14)	−0.0137 (14)
C19	0.0242 (16)	0.0229 (15)	0.0311 (15)	−0.0015 (12)	0.0072 (12)	−0.0076 (13)
C20	0.0250 (15)	0.0139 (13)	0.0169 (13)	−0.0004 (11)	0.0029 (11)	−0.0016 (11)
C21	0.0182 (14)	0.0167 (13)	0.0175 (13)	−0.0047 (11)	−0.0009 (11)	−0.0007 (11)
C22	0.0136 (15)	0.0359 (18)	0.048 (2)	−0.0057 (13)	0.0084 (13)	−0.0299 (16)
C23	0.0114 (13)	0.0225 (14)	0.0286 (15)	−0.0029 (11)	0.0009 (11)	−0.0135 (12)
C24	0.0182 (14)	0.0287 (15)	0.0205 (13)	−0.0029 (12)	−0.0017 (11)	−0.0118 (12)
C25	0.0153 (14)	0.0188 (13)	0.0193 (13)	−0.0010 (11)	−0.0021 (11)	−0.0061 (11)
C26	0.0112 (13)	0.0169 (13)	0.0228 (13)	0.0003 (10)	−0.0025 (10)	−0.0072 (11)
C27	0.0176 (14)	0.0205 (14)	0.0233 (14)	−0.0017 (11)	−0.0018 (11)	−0.0080 (11)
C28	0.0206 (15)	0.0325 (16)	0.0267 (15)	−0.0009 (12)	−0.0049 (12)	−0.0151 (13)
C29	0.0248 (16)	0.0410 (18)	0.0190 (14)	0.0022 (13)	−0.0064 (12)	−0.0073 (13)
C30	0.0190 (15)	0.0272 (15)	0.0234 (14)	0.0004 (12)	−0.0025 (12)	0.0003 (12)
C31	0.0135 (13)	0.0205 (14)	0.0211 (13)	0.0040 (11)	−0.0024 (11)	−0.0047 (11)
C32	0.0110 (13)	0.0167 (13)	0.0317 (15)	0.0002 (10)	0.0000 (11)	−0.0070 (12)
C33	0.0237 (15)	0.0168 (13)	0.0206 (13)	−0.0015 (11)	−0.0072 (11)	−0.0020 (11)
O1	0.0259 (11)	0.0282 (10)	0.0274 (10)	−0.0085 (8)	−0.0012 (8)	−0.0133 (8)
O2	0.0339 (11)	0.0203 (10)	0.0270 (10)	0.0040 (9)	−0.0069 (9)	−0.0139 (8)
O3	0.0240 (10)	0.0182 (10)	0.0325 (11)	0.0020 (8)	−0.0035 (8)	−0.0116 (8)
O4	0.0215 (11)	0.0226 (11)	0.0751 (15)	−0.0041 (9)	−0.0059 (10)	−0.0245 (11)
O5	0.0363 (13)	0.0581 (15)	0.0435 (13)	−0.0177 (11)	0.0053 (10)	−0.0381 (12)
O6	0.0359 (12)	0.0423 (12)	0.0222 (10)	−0.0133 (10)	−0.0075 (9)	−0.0143 (9)
O1W	0.0328 (11)	0.0330 (11)	0.0267 (10)	−0.0130 (9)	0.0071 (9)	−0.0159 (9)
O2W	0.0228 (10)	0.0384 (11)	0.0321 (11)	−0.0106 (9)	0.0054 (8)	−0.0237 (9)

### Geometric parameters (Å, º)

**Table:**

Cu1—N3	2.000 (2)	C10—H10A	0.9800
Cu1—N3^i^	2.000 (2)	C10—H10B	0.9800
Cu1—N1	2.017 (2)	C10—H10C	0.9800
Cu1—N1^i^	2.017 (2)	C11—O1	1.248 (3)
Cu1—O1w	2.5033 (19)	C11—O2	1.271 (3)
Cu1—O1w^i^	2.5033 (18)	C11—C12	1.501 (3)
Cu2—N4	1.9987 (19)	C12—C21	1.364 (3)
Cu2—N4^ii^	1.9987 (19)	C12—C13	1.431 (4)
Cu2—N6	2.0113 (19)	C13—O3	1.367 (3)
Cu2—N6^ii^	2.0114 (19)	C13—C14	1.383 (3)
Cu2—O2w	2.4681 (18)	C14—C15	1.424 (4)
Cu2—O2w^ii^	2.4681 (18)	C14—C33	1.514 (3)
N1—C4	1.479 (3)	C15—C16	1.423 (3)
N1—C1	1.491 (3)	C15—C20	1.426 (4)
N1—H1	1.0000	C16—C17	1.363 (4)
N2—C1	1.438 (3)	C16—H16	0.9500
N2—C2	1.442 (3)	C17—C18	1.406 (4)
N2—C5	1.455 (3)	C17—H17	0.9500
N3—C3	1.476 (3)	C18—C19	1.355 (4)
N3—C2	1.480 (3)	C18—H18	0.9500
N3—H3	1.0000	C19—C20	1.411 (4)
C4—C3^i^	1.518 (4)	C19—H19	0.9500
C4—H4A	0.9900	C20—C21	1.403 (3)
C4—H4B	0.9900	C21—H21	0.9500
C1—H1A	0.9900	C22—O4	1.245 (3)
C1—H1B	0.9900	C22—O5	1.279 (4)
C2—H2A	0.9900	C22—C23	1.507 (4)
C2—H2B	0.9900	C23—C32	1.366 (4)
C3—C4^i^	1.518 (4)	C23—C24	1.429 (4)
C3—H3A	0.9900	C24—O6	1.367 (3)
C3—H3B	0.9900	C24—C25	1.378 (3)
C5—H5A	0.9800	C25—C26	1.427 (3)
C5—H5B	0.9800	C25—C33	1.523 (3)
C5—H5C	0.9800	C26—C27	1.417 (3)
N6—C8	1.481 (3)	C26—C31	1.434 (3)
N6—C7	1.492 (3)	C27—C28	1.366 (4)
N6—H6	1.0000	C27—H27	0.9500
N4—C9	1.476 (3)	C28—C29	1.407 (4)
N4—C6	1.493 (3)	C28—H28	0.9500
N4—H4	1.0000	C29—C30	1.362 (4)
N5—C6	1.429 (3)	C29—H29	0.9500
N5—C7^ii^	1.432 (3)	C30—C31	1.418 (3)
N5—C10	1.460 (3)	C30—H30	0.9500
C7—N5^ii^	1.432 (3)	C31—C32	1.409 (3)
C7—H7A	0.9900	C32—H32	0.9500
C7—H7B	0.9900	C33—H33A	0.9900
C8—C9	1.518 (4)	C33—H33B	0.9900
C8—H8A	0.9900	O3—H3C	0.8400
C8—H8B	0.9900	O6—H6C	0.8400
C9—H9A	0.9900	O1W—H1WA	0.8641
C9—H9B	0.9900	O1W—H1WB	0.8606
C6—H6A	0.9900	O2W—H2WA	0.8578
C6—H6B	0.9900	O2W—H2WB	0.8629
			
N3—Cu1—N3^i^	180.00	H9A—C9—H9B	108.6
N3—Cu1—N1	93.47 (8)	N5—C6—N4	114.6 (2)
N3^i^—Cu1—N1	86.53 (8)	N5—C6—H6A	108.6
N3—Cu1—N1^i^	86.53 (8)	N4—C6—H6A	108.6
N3^i^—Cu1—N1^i^	93.47 (8)	N5—C6—H6B	108.6
N1—Cu1—N1^i^	180.0	N4—C6—H6B	108.6
N4—Cu2—N4^ii^	180.00	H6A—C6—H6B	107.6
N4—Cu2—N6	86.53 (8)	N5—C10—H10A	109.5
N4^ii^—Cu2—N6	93.47 (8)	N5—C10—H10B	109.5
N4—Cu2—N6^ii^	93.47 (8)	H10A—C10—H10B	109.5
N4^ii^—Cu2—N6^ii^	86.53 (8)	N5—C10—H10C	109.5
N6—Cu2—N6^ii^	180.0	H10A—C10—H10C	109.5
C4—N1—C1	112.6 (2)	H10B—C10—H10C	109.5
C4—N1—Cu1	105.83 (15)	O1—C11—O2	123.7 (2)
C1—N1—Cu1	115.85 (15)	O1—C11—C12	118.9 (2)
C4—N1—H1	107.4	O2—C11—C12	117.4 (2)
C1—N1—H1	107.4	C21—C12—C13	118.5 (2)
Cu1—N1—H1	107.4	C21—C12—C11	120.6 (2)
C1—N2—C2	115.5 (2)	C13—C12—C11	120.9 (2)
C1—N2—C5	114.8 (2)	O3—C13—C14	118.8 (2)
C2—N2—C5	113.9 (2)	O3—C13—C12	119.5 (2)
C3—N3—C2	112.89 (18)	C14—C13—C12	121.7 (2)
C3—N3—Cu1	106.69 (15)	C13—C14—C15	118.4 (2)
C2—N3—Cu1	115.45 (16)	C13—C14—C33	119.6 (2)
C3—N3—H3	107.1	C15—C14—C33	122.0 (2)
C2—N3—H3	107.1	C16—C15—C14	123.1 (2)
Cu1—N3—H3	107.1	C16—C15—C20	117.2 (2)
N1—C4—C3^i^	107.4 (2)	C14—C15—C20	119.7 (2)
N1—C4—H4A	110.2	C17—C16—C15	121.1 (3)
C3^i^—C4—H4A	110.2	C17—C16—H16	119.5
N1—C4—H4B	110.2	C15—C16—H16	119.5
C3^i^—C4—H4B	110.2	C16—C17—C18	120.9 (3)
H4A—C4—H4B	108.5	C16—C17—H17	119.5
N2—C1—N1	113.6 (2)	C18—C17—H17	119.5
N2—C1—H1A	108.8	C19—C18—C17	119.8 (3)
N1—C1—H1A	108.8	C19—C18—H18	120.1
N2—C1—H1B	108.8	C17—C18—H18	120.1
N1—C1—H1B	108.8	C18—C19—C20	121.0 (3)
H1A—C1—H1B	107.7	C18—C19—H19	119.5
N2—C2—N3	113.67 (19)	C20—C19—H19	119.5
N2—C2—H2A	108.8	C21—C20—C19	121.3 (2)
N3—C2—H2A	108.8	C21—C20—C15	118.8 (2)
N2—C2—H2B	108.8	C19—C20—C15	119.9 (2)
N3—C2—H2B	108.8	C12—C21—C20	122.1 (2)
H2A—C2—H2B	107.7	C12—C21—H21	118.9
N3—C3—C4^i^	107.57 (19)	C20—C21—H21	118.9
N3—C3—H3A	110.2	O4—C22—O5	124.0 (3)
C4^i^—C3—H3A	110.2	O4—C22—C23	119.0 (3)
N3—C3—H3B	110.2	O5—C22—C23	117.0 (3)
C4^i^—C3—H3B	110.2	C32—C23—C24	118.5 (2)
H3A—C3—H3B	108.5	C32—C23—C22	120.0 (2)
N2—C5—H5A	109.5	C24—C23—C22	121.4 (2)
N2—C5—H5B	109.5	O6—C24—C25	118.7 (2)
H5A—C5—H5B	109.5	O6—C24—C23	119.3 (2)
N2—C5—H5C	109.5	C25—C24—C23	122.0 (2)
H5A—C5—H5C	109.5	C24—C25—C26	119.0 (2)
H5B—C5—H5C	109.5	C24—C25—C33	119.4 (2)
C8—N6—C7	113.11 (19)	C26—C25—C33	121.5 (2)
C8—N6—Cu2	105.86 (15)	C27—C26—C25	123.1 (2)
C7—N6—Cu2	115.23 (15)	C27—C26—C31	117.6 (2)
C8—N6—H6	107.4	C25—C26—C31	119.2 (2)
C7—N6—H6	107.4	C28—C27—C26	121.1 (2)
Cu2—N6—H6	107.4	C28—C27—H27	119.4
C9—N4—C6	113.30 (18)	C26—C27—H27	119.4
C9—N4—Cu2	106.73 (14)	C27—C28—C29	120.9 (2)
C6—N4—Cu2	116.27 (15)	C27—C28—H28	119.6
C9—N4—H4	106.6	C29—C28—H28	119.6
C6—N4—H4	106.6	C30—C29—C28	120.0 (2)
Cu2—N4—H4	106.6	C30—C29—H29	120.0
C6—N5—C7^ii^	115.2 (2)	C28—C29—H29	120.0
C6—N5—C10	116.4 (2)	C29—C30—C31	120.7 (2)
C7^ii^—N5—C10	114.1 (2)	C29—C30—H30	119.6
N5^ii^—C7—N6	113.9 (2)	C31—C30—H30	119.6
N5^ii^—C7—H7A	108.8	C32—C31—C30	121.4 (2)
N6—C7—H7A	108.8	C32—C31—C26	119.0 (2)
N5^ii^—C7—H7B	108.8	C30—C31—C26	119.5 (2)
N6—C7—H7B	108.8	C23—C32—C31	122.0 (2)
H7A—C7—H7B	107.7	C23—C32—H32	119.0
N6—C8—C9	107.5 (2)	C31—C32—H32	119.0
N6—C8—H8A	110.2	C14—C33—C25	115.6 (2)
C9—C8—H8A	110.2	C14—C33—H33A	108.4
N6—C8—H8B	110.2	C25—C33—H33A	108.4
C9—C8—H8B	110.2	C14—C33—H33B	108.4
H8A—C8—H8B	108.5	C25—C33—H33B	108.4
N4—C9—C8	107.08 (19)	H33A—C33—H33B	107.4
N4—C9—H9A	110.3	C13—O3—H3C	109.5
C8—C9—H9A	110.3	C24—O6—H6C	109.5
N4—C9—H9B	110.3	H1WA—O1W—H1WB	114.2
C8—C9—H9B	110.3	H2WA—O2W—H2WB	113.5
			
C1—N1—C4—C3^i^	169.94 (19)	C18—C19—C20—C15	−3.3 (4)
Cu1—N1—C4—C3^i^	42.4 (2)	C16—C15—C20—C21	−175.3 (2)
C2—N2—C1—N1	68.3 (3)	C14—C15—C20—C21	3.3 (3)
C5—N2—C1—N1	−67.3 (3)	C16—C15—C20—C19	3.2 (3)
C4—N1—C1—N2	−177.0 (2)	C14—C15—C20—C19	−178.3 (2)
Cu1—N1—C1—N2	−55.0 (2)	C13—C12—C21—C20	−5.8 (3)
C1—N2—C2—N3	−69.9 (3)	C11—C12—C21—C20	174.5 (2)
C5—N2—C2—N3	66.1 (3)	C19—C20—C21—C12	−174.1 (2)
C3—N3—C2—N2	−179.3 (2)	C15—C20—C21—C12	4.4 (3)
Cu1—N3—C2—N2	57.6 (2)	O4—C22—C23—C32	4.7 (4)
C2—N3—C3—C4^i^	−169.0 (2)	O5—C22—C23—C32	−175.6 (2)
Cu1—N3—C3—C4^i^	−41.1 (2)	O4—C22—C23—C24	−174.3 (2)
C8—N6—C7—N5^ii^	179.2 (2)	O5—C22—C23—C24	5.4 (4)
Cu2—N6—C7—N5^ii^	57.3 (3)	C32—C23—C24—O6	179.7 (2)
C7—N6—C8—C9	−169.3 (2)	C22—C23—C24—O6	−1.2 (4)
Cu2—N6—C8—C9	−42.3 (2)	C32—C23—C24—C25	−1.5 (4)
C6—N4—C9—C8	−171.1 (2)	C22—C23—C24—C25	177.6 (2)
Cu2—N4—C9—C8	−41.8 (2)	O6—C24—C25—C26	−176.4 (2)
N6—C8—C9—N4	57.0 (3)	C23—C24—C25—C26	4.8 (4)
C7^ii^—N5—C6—N4	−67.8 (3)	O6—C24—C25—C33	0.5 (4)
C10—N5—C6—N4	69.6 (3)	C23—C24—C25—C33	−178.3 (2)
C9—N4—C6—N5	178.7 (2)	C24—C25—C26—C27	172.3 (2)
Cu2—N4—C6—N5	54.5 (3)	C33—C25—C26—C27	−4.5 (4)
O1—C11—C12—C21	−1.3 (3)	C24—C25—C26—C31	−4.7 (4)
O2—C11—C12—C21	178.1 (2)	C33—C25—C26—C31	178.5 (2)
O1—C11—C12—C13	178.9 (2)	C25—C26—C27—C28	−178.4 (2)
O2—C11—C12—C13	−1.6 (3)	C31—C26—C27—C28	−1.3 (4)
C21—C12—C13—O3	−179.3 (2)	C26—C27—C28—C29	−0.8 (4)
C11—C12—C13—O3	0.5 (3)	C27—C28—C29—C30	1.8 (4)
C21—C12—C13—C14	−0.5 (3)	C28—C29—C30—C31	−0.5 (4)
C11—C12—C13—C14	179.2 (2)	C29—C30—C31—C32	176.6 (2)
O3—C13—C14—C15	−173.3 (2)	C29—C30—C31—C26	−1.7 (4)
C12—C13—C14—C15	7.9 (3)	C27—C26—C31—C32	−175.8 (2)
O3—C13—C14—C33	5.4 (3)	C25—C26—C31—C32	1.4 (3)
C12—C13—C14—C33	−173.4 (2)	C27—C26—C31—C30	2.6 (3)
C13—C14—C15—C16	169.3 (2)	C25—C26—C31—C30	179.7 (2)
C33—C14—C15—C16	−9.4 (3)	C24—C23—C32—C31	−2.1 (4)
C13—C14—C15—C20	−9.2 (3)	C22—C23—C32—C31	178.9 (2)
C33—C14—C15—C20	172.1 (2)	C30—C31—C32—C23	−176.3 (2)
C14—C15—C16—C17	−179.0 (2)	C26—C31—C32—C23	2.1 (4)
C20—C15—C16—C17	−0.5 (3)	C13—C14—C33—C25	119.6 (3)
C15—C16—C17—C18	−2.1 (4)	C15—C14—C33—C25	−61.7 (3)
C16—C17—C18—C19	2.0 (4)	C24—C25—C33—C14	120.9 (3)
C17—C18—C19—C20	0.7 (4)	C26—C25—C33—C14	−62.3 (3)
C18—C19—C20—C21	175.1 (2)		

Symmetry codes: (i) −*x*+2, −*y*+1, −*z*+1; (ii) −*x*+1, −*y*+1, −*z*.

### Hydrogen-bond geometry (Å, º)

**Table:**

*D*—H···*A*	*D*—H	H···*A*	*D*···*A*	*D*—H···*A*
N1—H1···O3^iii^	1.00	2.50	3.272 (3)	134
N3—H3···O5	1.00	1.89	2.836 (3)	156
N4—H4···O2^iii^	1.00	1.90	2.822 (3)	152
O3—H3*C*···O2	0.84	1.75	2.502 (2)	148
O6—H6*C*···O5	0.84	1.75	2.514 (3)	150
O1*W*—H1*WA*···O1^iv^	0.86	1.88	2.746 (2)	178
O1*W*—H1*WB*···O4	0.86	2.31	3.136 (3)	162
O1*W*—H1*WB*···O5	0.86	2.41	3.087 (3)	136
O2*W*—H2*WA*···O1^iii^	0.86	2.05	2.901 (2)	169
O2*W*—H2*WA*···O2^iii^	0.86	2.61	3.280 (2)	136
O2*W*—H2*WB*···O4^v^	0.86	1.88	2.743 (3)	176
C2—H2*B*···O1^vi^	0.99	2.48	3.435 (3)	162
C5—H5*B*···O2^iii^	0.98	2.45	3.316 (3)	147

Symmetry codes: (iii) *x*, *y*+1, *z*; (iv) *x*+1, *y*+1, *z*; (v) *x*−1, *y*, *z*; (vi) −*x*+1, −*y*, −*z*+1.
